# eMZed: an open source framework in Python for rapid and interactive development of LC/MS data analysis workflows

**DOI:** 10.1093/bioinformatics/btt080

**Published:** 2013-02-15

**Authors:** Patrick Kiefer, Uwe Schmitt, Julia A. Vorholt

**Affiliations:** ^1^ETH Zurich, Institute of Microbiology, 8093 Zurich, Switzerland and ^2^mineway GmbH, 66121 Saarbrücken, Germany

## Abstract

**Summary:** The Python-based, open-source eMZed framework was developed for mass spectrometry (MS) users to create tailored workflows for liquid chromatography (LC)/MS data analysis. The goal was to establish a unique framework with comprehensive basic functionalities that are easy to apply and allow for the extension and modification of the framework in a straightforward manner. eMZed supports the iterative development and prototyping of individual evaluation strategies by providing a computing environment and tools for inspecting and modifying underlying LC/MS data. The framework specifically addresses non-expert programmers, as it requires only basic knowledge of Python and relies largely on existing successful open-source software, e.g. OpenMS.

**Availability:** The framework eMZed and its documentation are freely available at http://emzed.biol.ethz.ch/. eMZed is published under the GPL 3.0 license, and an online discussion group is available at https://groups.google.com/group/emzed-users.

**Contact:** kiefer@micro.biol.ethz.ch

**Supplementary information:** Supplementary data are available at *Bioinformatics* online.

## 1 INTRODUCTION

Liquid chromatography/mass spectrometry (LC/MS) data analysis generally requires flexible software tools. Although a number of solutions for specific or multiple applications currently exist, many of these belong to one of two extremes. The first group includes frameworks that are highly flexible but have been developed in languages (e.g. C++) that require advanced programming skills, e.g. OpenMS (Sturm *et al.*, 2008). Although such frameworks have a rapid application run time, the testing of new workflows and concepts is cumbersome because programming requirements are high, and edit-compile cycles are slow. The second group includes closed black-box solutions with graphical user interfaces that are easy to use but inherently non-transparent and inflexible, e.g. Maven (Melamud *et al.*, 2010) and mzMine2 (Pluskal *et al.*, 2010). Note that libraries such as the R-based XCMS (Smith *et al.*, 2006) or the Matlab-based Bioinformatics Toolbox (Mathworks, Natick, MA, USA) lie between these extremes. The motivation to develop eMZed was to provide an open-source framework to establish transparent and flexible workflows for high-end data treatment that requires only basic programming skills of the user. To this end, we combined the powerful and easy-to-learn programming language Python, a comprehensive library of elementary building blocks, and an integrated development environment.

## 2 RESULTS

### 2.1 Technical aspects

The eMZed framework is implemented in the Python programming language, which is well established in scientific computing (Oliphant, 2007) and bioinformatics in particular (Cock *et al.*, 2009). Compared with R and Matlab, Python’s standard library is more extensive and enables rapid application development by various means; e.g. Python supports easy access to online services such as PubChem or Metlin, which are of great interest for metabolomics data analyses. We used the Python libraries PyQt4, spyderlib and guiqwt to build the workbench and graphical explorers and used numpy and scipy for the numerical data structures and algorithms.

One central concept in the development of eMZed was the integration of previously established algorithms into a single platform that minimizes error-prone import and export steps. Therefore, we integrated functionalities from the libraries XCMS and OpenMS. To call functionalities from XCMS, we built a bridge to R that enables eMZed to use the centWave feature detector (Tautenhahn *et al.*, 2008) and ‘matched filter’ method (Smith *et al.*, 2006). Enabling access to a subset of OpenMS functionalities for fast I/O and providing clustering-based retention time alignment (Lange *et al.*, 2007) represented a major obstacle that was overcome by developing a code generator. This generator is hosted at https://github.com/uweschmitt/pyOpenMS and uses Cython for invoking C/C++ functions.

The current version of eMZed was developed and tested using 32-bit Windows 7 and was further tested using 64-bit Ubuntu 12.04 Linux. A 64-bit version for Windows is currently being developed.

### 2.2 Functionalities

eMZed provides simple and readily usable building blocks for rapid workflow development. In addition to data inspection, peak detection, alignment and integration, the current version possesses several dedicated helper modules that support the building of graphical dialogues, statistical analyses and chemical data examinations, such as mass and isotope abundance analyses and the manipulation of molecular formulas, for example. For peak identification, access to the chemical compound database PubChem (Bolton *et al.*, 2008) and the Metlin online service (Smith *et al.*, 2005) is provided.

**Fig. 1.**
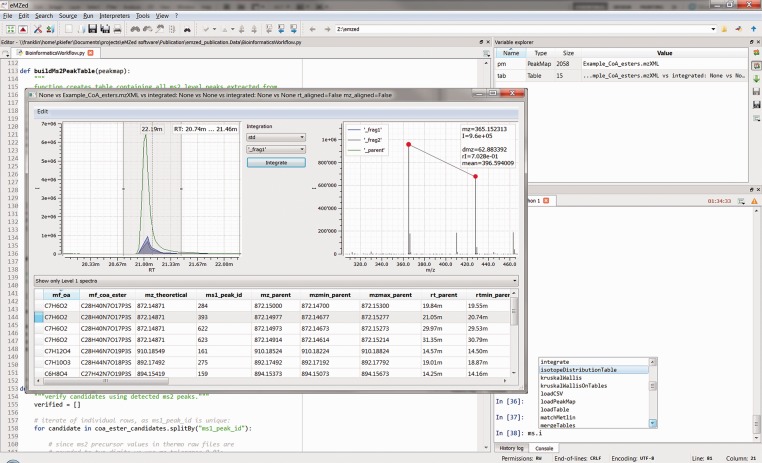
Screenshot of the eMZed workbench showing the editor, variable explorer, IPython console and interactive table explorer. The table explorer shows the results of a coenzyme A ester identification workflow (see Supplementary Material). Peaks of the parent ion and integrated peaks of two fragment ions are depicted in the left plot. The right plot shows a corresponding MS level 2 spectrum that includes information on selected m/z peaks (red dots)

LC/MS data are handled using PeakMap and Spectrum data structures, and interactive explorer tools are linked to these data structures for visual data inspection. Table is a comprehensive data structure supporting SQL-like operations. Table plays a key role in eMZed workflows because it provides easy handling of peaks or chemical data and supports the identification and integration of MS level 1 and level 2 peaks. Note that chromatographic peaks and spectra can also be directly visualized within Table structure (Fig. 1). In addition, Table can be edited, thereby allowing for the modification of peak and integration limits or the deletion and duplication of rows. PeakMap and Table are available in the workspace variable explorer, and interactive inspection can be integrated into workflows to validate intermediate or final results. A complete overview of all features can be found at the eMZed homepage.

### 2.3 Example application

To demonstrate the comprehensive functionalities of eMZed, we implemented a tailored workflow for the database-independent identification of coenzyme A thioesters of MS level 1 and level 2 spectra. The workflow can be subdivided into four steps:

- Creation of a coenzyme A ester solution space from a restricted recombination of chemical elements C, H, N, O, P and S.
- Detection of high-resolution MS level 1 peaks using the centWave feature detector and the identification of candidates using the Table join operation.
- Evaluation of candidates by comparing m/z values of measured MS level 2 peaks with values of specific fragment ions calculated from assigned molecular formulas.
- Visualization of a result table for inspection.

The given example demonstrates that even complex operations can be encoded easily owing to the multitude of functionalities that are available. A more detailed description of the workflow, the Python code and example data are provided in the Supplementary Material.

## 3 DISCUSSION

Metabolomics and related fields are rapidly progressing and require the development and modification of workflows and analytical strategies. In this context, the speed of data analysis routines is an important factor, although efforts to implement and test new solutions are equally important. To this end, eMZed provides a workspace and capability to inspect and visualize interim results at each step of data processing. In addition, eMZed provides a common base for developing individual applications and supports interchangeable individual solutions. This approach may help to simplify the current landscape of existing LC/MS software, which is fragmented and often laboratory specific.

## 4 OUTLOOK

Future work will be directed towards the implementation of new features, which, e.g. will allow for enhanced MS level 2 data handling, port eMZed to 64 bit Windows 7 operating system, better support of R and faster analysis by multi core support. These enhancements will be available in forthcoming versions of eMZed.

*Funding*: This project was support by ETH Zurich, Department of Biology, within the frame of an IT-strategy initiative. Complementary funding was obtained via the Swiss Initiative in Systems Biology SystemsX.ch, BattleX.

*Conflict of interest*: none declared.

## Supplementary Material

Supplementary Data
