# Supplementary material for: eMZed: an open source framework in Python for rapid and interactive development of LC/MS data analysis workflows
Source: Bioinformatics. 2013 Feb 15;29(7):963–4. doi: 10.1093/bioinformatics/btt080 (PMC3605603; doi:10.1093/bioinformatics/btt080)
Supplement: Supplementary Data [file supp_29_7_963__index.html]

eMZed: an open source framework in Python for rapid and interactive development of LC/MS data analysis workflows — eMZed: an open source framework in Python for rapid and interactive development of LC/MS data analysis workflows — Supplementary Data 

# eMZed: an open source framework in Python for rapid and interactive development of LC/MS data analysis workflows

## Supplementary Data

files

**Files in this Data Supplement:**

- Supplementary Data - doc file
