# Supplementary material for: eMZed: an open source framework in Python for rapid and interactive development of LC/MS data analysis workflows
Source: Bioinformatics. 2013 Feb 15;29(7):963–4. doi: 10.1093/bioinformatics/btt080 (PMC3605603; doi:10.1093/bioinformatics/btt080)
Supplement: Supplementary Data [file supp_btt080_Bioinf2012-0981Kiefer_supplementary_material.doc]

# Supplementary Material

Example workflow “Identification of coenzyme A thioesters”

**Introduction**

Coenzyme A thioesters (CoA esters) play an important role in a multitude of metabolic pathways. Besides fatty acid metabolism they are involved in essential pathways of *Procaryotes* i.e. alternative pathway of glyoxylate regeneration (, ) or anaerobic degradation of benzoate . Often these pathway intermediates are unknown and not part of a database. We therefore developed a strategy to identify those CoA esters *ab initio* : A solution space of potential CoA ester molecular formulas can be created from a restricted recombination of elements C,H,N,O,P and S. Since CoA esters form mainly [M+H]+ ions with applied LC-MS method an inclusion list of potential parent ions build from CoA solution space was used for targeted fragmentation of protonated CoA ester candidates. To identify CoA esters on MS 2 level, fragment spectra of selected parent ions can be searched for presence of two distinct fragment ions : A CoA-specific fragment ion at m/z = 428.0367 remaining unchanged for all CoA esters and a characteristic fragment ion arising from the molecular region containing the CoA esters that allows determining the elemental composition of the esterified organic acid when using high-resolution mass spectrometry for fragment ion analysis. The mass of this fragment can be calculated from the molecular formula by subtracting a common neutral loss during fragmentation procedure.

**Description**

We implemented following workflow using eMZed that allows automatic *ab initio* identification of CoA esters on MS level 1 and 2 including following steps:

1. Creating a CoA ester solution space (function “buildCoaSolutionSpace”) as table data type.
2. Detecting high resolution MS level 1 peaks using the centWave feature detector for high resolution mass spectrometry data (function “detectPeaks”) and identifying potential CoA esters using the table join operation (function “identifyCoaEster”)
3. Extracting MS level 2 peaks and converting them into table data type (function “buildMs2PeakTable”). Since MS level 2 data acquisition was based on inclusion list strategy of the LTQ Orbitrap instrument we could not use centWave. We therefore performed MS level 2 peak detection via targeted extraction of fragment ion peaks by fitting an EMG model based on *a priori* known bounds for masses and retention times (function “detectMs2FragmentPeaks”).
4. Evaluating CoA ester candidates on MS level 2 by calculating mz values of specific fragment ions from CoA ester molecular formula (function “neutralFragmentMasses”) and checking for their presence in MS level 2 data (function “verifyCandidates”).

The results are presented as editable table (Fig. 1) presenting MS level 1 and 2 data for each CoA ester, which allows individual inspection of identified CoA ester peaks and further modification of the results.

The total script size without comments and empty lines is 104 lines.

We included an example data file from LC-MS/MS analysis of CoA ester extracts of *Thauera chlorobenzoica* grown on benzoate as sole carbon and energy. LC-MS/MS analysis was carried out on an LTQ Orbitrap instrument as described previously (Kuntze, *et al.*, 2011).

For given example total runtime was 7.4 s on 64 bit Windows 7 system, Processor Intel(R) Core™ i7 2640M CPU @ 2.80GHz 2.80 GHz.

A zipped file containing example data and python script can be downloaded at <http://www.micro.biol.ethz.ch/downloads/eMZed>

**Running Workflow**


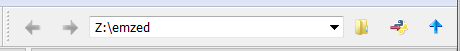


1.1

2.1

1) Press the "choose folder button" of eMZed taskbar (1.) and choose directory with downloaded demo workflow BioinformaticsWorkflow.py

2) Press "set button" (2.) to set the current working directory to the chosen one

3) Choose in top menu "File->Open" and open "BioinformaticsWorkflow.py"

4) Press F5 to execute this file in the IPython shell (with the first execution a configuration dialog will open: Choose "execute in current Python or IPython interpreter" and press "Run")

5) After the workflow is executed a dialog showing the results is opened. If your OS is Windows, the dialog might be opened in the background. In this case check the taskbar and activate this dialog.

6) You can inspect the identified peaks by clicking left to a row, zooming in the two plots is done by pressing the right mouse button while moving the mouse.

Daluge, J.J.*, et al.* (2002) Separation and identification of organic acid-coenzyme A thioesters using liquid chromatography/electrospray ionization-mass spectrometry *Anal Bioanal Chem*, **374**, 835-840.

Erb, T.J.*, et al.* (2007) Synthesis of C5-dicarboxylic acids from C2-units involving crotonyl-CoA carboxylase/reductase: the ethylmalonyl-CoA pathway, *Proc Natl Acad Sci U S A*, **104**, 10631-10636.

Kuntze, K.*, et al.* (2011) Enzymes involved in the anaerobic degradation of meta-substituted halobenzoates, *Mol Microbiol*, **82**, 758-769.

Peyraud, R.*, et al.* (2009) Demonstration of the ethylmalonyl-CoA pathway by using 13C metabolomics, *Proc Natl Acad Sci U S A*, **106**, 4846-4851.

Wischgoll, S.*, et al.* (2005) Gene clusters involved in anaerobic benzoate degradation of Geobacter metallireducens, *Mol Microbiol*, **58**, 1238-1252.

**APPENDIX: Code**

import mass

import ms

__doc__ = """

This demo workflow is part of the supplementary material to the

bioinformatics article about eMZed.

More information about eMZed at http://emzed.ethz.ch

Copyright 2012 by Patrick Kiefer and Uwe Schmitt.

This code is free software: you can redistribute it and/or modify it

under the terms of version 3 of the GNU General Public License as

published by the Free Software Foundation.

"""

def **runCoaAnalysi**s(peakmap, max_carbon_count):

"""

1) detect ms1 peaks in peakmap.

2) compare with theoretical peaks of coa esters from calculated solution space,

where max_carbon_count is the number of carbon atoms of esterified organic acids.

3) verify these peaks using ms2 fragments.

"""

# detect peaks

ms1_peaks = detectPeaks(peakmap)

# build solution Space of potential CoA esters within Mass range

mzmax = max(spec.mzMax() for spec in peakmap.levelNSpecs(1))

sol_space = buildCoaSolutionSpace(max_carbon_count, mzmax)

# find matching mz pairs from solution space and detected peaks

coa_ester_candidates = identifyCoaEster(sol_space, ms1_peaks)

# identify these candidates using ms2 data:

ms2_peaks = buildMs2PeakTable(peakmap)

identified = verifyCandidates(coa_ester_candidates, ms2_peaks)

result = cleanupTableColumns(identified)

return result

def **detectPeaks**(peakmap):

"""run centwave feature detector for high resolution mass

spectrometry data and do some postprocessing of mz windows

"""

t = ms.runCentwave(peakmap, ppm=20, peakwidth=(12, 60),

prefilter=(1, 500), snthresh = 0.1, mzdiff=0.001)

# using the parameters above centwave returns mz ranges sometimes including

# only a part of peak spectra, which result in irregular peaks, so we widen

# up this range:

mz_tolerance = 3 * MMU

t.replaceColumn("mzmin", t.mz - mz_tolerance)

t.replaceColumn("mzmax", t.mz + mz_tolerance)

return t

def **buildCoaSolutionSpace**(max_carbon_count, mz_max):

""" A CoA ester solution space is built by creating mf of possible

organic acids using the formula table creator based on HR2 formual

generator from http://fiehnlab.ucdavis.edu/projects/Seven_Golden_Rules/.

Those organic acids with maximum number of carbon atoms max_carbon_count are

esterified with coenzyme A.

"""

# calculate the upper limit of organic acids molecular weight depending

# on acquisition mass range:

coa = "C21H36N7O16P3S"

m0_max_acid = mz_max - mass.of(coa) + mass.of("H2O")

m0_min_acid = mass.of("HCOOH") # smallest organic acid, formic acid

# start with generation of possible organic acids:

sol_space = ms.formulaTable(m0_min_acid, m0_max_acid,

C=(1, max_carbon_count), O=(2, None),

H=(2, None), N=0, S=0, P=0)

sol_space.renameColumns(mf="mf_oa", m0="m0_oa")

# build coa-ester formula:

def **addEster**(mf_oa):

return ms.addmf(mf_oa, coa, "-H2O")

sol_space.addColumn("mf_coa_ester", sol_space.mf_oa.apply(addEster))

# add neutral masses of coa ester:

sol_space.addColumn("m0_coa_ester", sol_space.mf_coa_ester.apply(mass.of))

# mz of [M+H]+ adduct

sol_space.addColumn("mz_theoretical", sol_space.m0_coa_ester + mass.p)

return sol_space

def **identifyCoaEster**(sol_space, ms1_peaks):

""" Matches theoretical mz values from solution space and found ms 1 peaks.

"""

# sql like join combines tables sol_space and ms1_peaks such that

# mz values match up to given tolerance:

coa_ester_candidates = sol_space.join(ms1_peaks,

ms1_peaks.mz.approxEqual(sol_space.mz_theoretical, 5 * MMU))

# the join above generated column id__0 which we rename for better

# understanding:

coa_ester_candidates.renameColumns(id__0="ms1_peak_id")

return coa_ester_candidates

def **buildMs2PeakTable**(peakmap):

""" function creates table containing all ms2 level peaks extracted from

data_set.

"""

from libms.DataStructures.MSTypes import PeakMap

# we split ms2 spectra in peakmap. the following method returns a

# mapping pre_cursor_mz --> [ spec1, .... ]:

ms2_spectra = peakmap.splitLevelN(2, significant_digits_precursor=2)

tables = []

for pre_mz, spectra in ms2_spectra.items():

# we build a table with one row in each iteration. each table

# represents a peak on ms 2 level.

# start with single column table with one row:

ms2_peakmap = PeakMap(spectra)

t = ms.toTable("precursor", [pre_mz])

# add rt and mz ranges so that table explorer can visualize

# total ion chormatograms (TICs)

rtmin, rtmax = ms2_peakmap.rtRange()

t.addColumn("rtmin", rtmin, type_=float)

t.addColumn("rtmax", rtmax, type_=float)

mzmin, mzmax = ms2_peakmap.mzRange()

t.addColumn("mzmin", mzmin, type_=float)

t.addColumn("mzmax", mzmax, type_=float)

# add underlying ms2 spectra

t.addColumn("peakmap", ms2_peakmap)

tables.append(t)

# merge list of tables to one table:

ms2_peaks = ms.mergeTables(tables)

# add id-column with unique id for each row:

ms2_peaks.addEnumeration('ms2_peak_id')

return ms2_peaks

def **verifyCandidates**(coa_ester_candidates, ms2_peaks):

""" verify candidates using detected ms2 peaks """

verified = []

# iterate of individual rows, as ms1_peak_id is unique:

for candidate in coa_ester_candidates.splitBy("ms1_peak_id"):

# since ms2 precursor values in thermo raw files are

# rounded to two digits we use mz tolerance 0.01:

precursor_matches = ms2_peaks.filter(

ms2_peaks.precursor.approxEqual(candidate.mz_theoretical, 0.01))

for precursor in precursor_matches.splitBy("ms2_peak_id"):

# extract some values from candidate table

mf_coa_ester = candidate.mf_coa_ester.uniqueValue()

rtmin_parent = candidate.rtmin__0.uniqueValue(up_to_digits=2)

rtmax_parent = candidate.rtmax__0.uniqueValue(up_to_digits=2)

# theoretical fragment masses for given molecular formula

fragment_masses = neutralFragmentMasses(mf_coa_ester)

# detect these fragments

fragments = detectMs2FragmentPeaks(precursor, fragment_masses,

rtmin_parent, rtmax_parent)

# both fragment ions detected?

if len(fragments) == 2:

# now combine these two lines to one line, which eases

# inspection

first_frag, second_frag = fragments.splitBy("mz_frag")

subtable = candidate.join(first_frag).join(second_frag)

verified.append(subtable)

return ms.mergeTables(verified)

def **neutralFragmentMasses**(mf_coa_ester):

"""

common fragments of CoA Esters described by

Daluge, J. J., S. Gort, et al. (2002).

"Separation and identification of organic acid-coenzyme A thioesters

using liquid chromatography/electrospray ionization-mass spectrometry"

Anal Bioanal Chem 374: 835-840.

"""

neutral_loss_mf = "C10H16N5O13P3"

common_mf = "C10H15N5O10P2"

return [mass.of(mf_coa_ester) - mass.of(neutral_loss_mf), mass.of(common_mf)]

def **detectMs2FragmentPeaks**(ms2_peak_table, fragment_masses, rtmin_parent, rtmax_parent):

""" Integration of ms level 2 peaks. The resuluting peak area is

used for detection of coa ester specific fragment ion peaks.:

we do not use centwave here, because most of our ms2 peaks do not form

rerular chromatograms. so we just integrate the theoretic range and filter

for non-zero area.

"""

mz_tol = 10 * MMU # mass accuracy of ms 2 data

# we build a table where each row represents a fragment:

fragment_table = ms.toTable("m0_frag", fragment_masses)

# calculate [M+H]+ adducts mass:

fragment_table.addColumn("mz_frag", fragment_table.m0_frag + mass.p)

# full join, that is cross product of ms2_peak_table and

# fragment_table:

ms2_peak_table = ms2_peak_table.join(fragment_table)

# the join above generated column mz_frag__0 which we rename for

# easier reading:

ms2_peak_table.renameColumns(mz_frag__0 = "mz_frag")

# replaces ranges for TICs by ranges for fragment peaks:

ms2_peak_table.replaceColumn("mzmin", ms2_peak_table.mz_frag - mz_tol)

ms2_peak_table.replaceColumn("mzmax", ms2_peak_table.mz_frag + mz_tol)

ms2_peak_table.replaceColumn("rtmin", rtmin_parent)

ms2_peak_table.replaceColumn("rtmax", rtmax_parent)

# calculates representing mz value for peaks in given rt/mz range:

ms.recalculateMzPeaks(ms2_peak_table)

# detect ms2 peaks by integration

ms2_peak_table = ms.integrate(ms2_peak_table, "trapez", msLevel=2)

detected = ms2_peak_table.filter(ms2_peak_table.area > 0)

return detected

def **cleanupTableColumns**(table):

""" clean up column names in the resulting table """

table.dropColumns('centwave_config__0', 'intb__0', 'into__0', 'm0_coa_ester',

'm0_frag__2', 'm0_frag__4', 'm0_oa', 'maxo__0',

'ms2_peak_id__1', 'ms2_peak_id__3', 'polarity__0',

'precursor__3', 'sample__0', sn__0', 'source__0')

table.renameColumns(precursor__1="precursor")

table.renamePostfixes(__0="_parent", __1="_frag1", __3="_frag2")

table.renameColumns(mz_frag_frag2="mz_theoretical_frag2",

mz_frag_frag1="mz_theoretical_frag1")

return table

# Start workflow with example

if __name__ == "__main__":

pm = ms.loadPeakMap("Example_CoA_esters.mzXML")

tab = runCoaAnalysis(pm, 100)

print "\nWORKFLOW FINISHED !"

ms.inspect(tab)
